# From the Collective Unconscious to the Narrative Unconscious: Re-Imagining the Sources of Selfhood

**DOI:** 10.5964/ejop.v12i4.1325

**Published:** 2016-11-18

**Authors:** Mark Freeman

**Affiliations:** a College of the Holy Cross, Worcester, MA, USA

## The Shock of History

During the course of a symposium at the most recent conference on the dialogical self, a question was posed in which Jung’s idea of the collective unconscious was brought to bear on the matters being discussed. That idea has never had much currency in the more traditional quarters of psychological inquiry. Like much of Jung’s work, it hints of a kind of mysticism most academic psychologists are reluctant to entertain. Worse still, it also hints of a kind of *volk*-inspired way of thinking that, according to some (e.g., Noll, 1994), is a too-short step to Nazism and other such mythological fetishizations of the psyche. At least partially valid though some of these criticisms are, it’s unfortunate that the idea of the collective unconscious has met with the rather icy reception it has. My main reason for saying so is that there are dimensions of experience—religious experience and aesthetic experience, especially—that point in the direction of a kind of ecstatic primordiality that appears difficult to account for *without* invoking something akin to the Jungian idea. What’s more, the quite extraordinary continuities of symbolism, in religion, mythology, and across the course of history more generally would seem to suggest that the idea isn’t as farfetched as many a modern-day psychologist assumes.

Having said all this, I was quick to distinguish Jung’s idea of the collective unconscious from what I have come to call the *narrative* unconscious (see, e.g., Freeman, 2001, 2002). The idea began with an extremely intense experience I had the first time I was in Berlin, which I described as follows (in Freeman, 2002):

Although I approached visiting Berlin with some measure of uncertainty over how I would respond to being there—given my own Jewish background, the books I had read, the movies I had seen, and so on—I didn’t experience any particular intensity about the prospect. For one, the first few days were to be spent at an academic conference, where the topics had little to do with Germany and its past. For another, I would be with friends who lived in Berlin and were eager to show me around, share some food and drink, and have a good time. As it turned out, the conference was in fact uneventful as were the first couple of days touring the city. We saw lots of sights, we shared some meals, and, in the face of all the cranes dotting the landscape, we talked a good deal about the challenges the city faced as it sought to rebuild itself, literally and figuratively. It was all fascinating to me, but in a somewhat distanced way. There were emotions, to be sure, but they were blunted and rather generic: “How terrible it must have been.” “It’s all so hard to imagine.” “It’s incredible how fresh the wounds still seem to be, all those buildings, pieces ripped out of them by shells, the commemorative plaques, the armed guards in front of synagogues, even now.” And so forth and so on.

But then something strange, and utterly unexpected, happened. I still don’t know quite how to make sense of it. I was traveling on a bus through the city when everything that had been at a distance suddenly came near: the cranes, the buildings, the gardens, the Brandenburg Gate, the Reichstag. Everything that had been a fascinating or disturbing spectacle, an object to be beheld, taken in—as when one takes in the sights of any city—had become a kind of living, breathing presence. When I initially tried to explain this to someone, I said that I had never had such an intense experience of *history* as I had had then, during those moments.

As for my response to this sudden transformation from spectacle to presence, it was something like a deep grief, a mixture of sorrow and horror, rolled into one. I was either weeping or on the verge of it for a good amount of time afterward. It was very strange and very powerful. Lest this episode be construed as an index of my emotional stability, I am not given to this sort of thing. There was, in other words, something truly extra-ordinary about the entire set of experiences. It was extremely disturbing. (pp. 196-197)

The question, of course, was: What had happened? Initially, I found myself entertaining “an almost mystical path . . . as if death was in the air,” and “I couldn’t help but wonder whether it was possible for the events of the past—terrible ones, in particular—to somehow leave traces, in the form of disturbed energy fields or some such thing.” I wondered whether “the past could somehow become inscribed in the present,” whether “it remained alive and operative. . . . Would someone who stumbled upon a piece of land where a concentration camp once stood, without knowing where he or she was, feel anything different? Would there be traces or echoes, even *ghosts* of a sort? It’s possible.” It’s also possible that what I was beholding somehow tapped into ancient memory, Jung-style, and activated some sort of archetypal image of death and destruction, one that was palpable enough to send me reeling. “But,” I had written, “the modern mind—including my own—finds it difficult to move into regions like these, intriguing though they are” (p. 198).

I wouldn’t be one to rule out either of these ideas. They don’t easily square with the way most of us have come to think about the world. And, they are speculative in a way that can’t help but make the empirically-oriented psychologist ontologically and epistemologically squeamish. *Could* there be weird energy fields in the world, deposited by the awful crimes of the past? *Could* there be a collective unconscious, waiting, as it were, for an encounter like this one to kick it into gear? Who knows? These possibilities were enticing then and remain so now. “But then I had to pull back a bit. Somehow or other,” I ventured, “I must have ‘brought’ things with me that set in motion the experiences of the day. That is to say, I must have been hermeneutically ‘prepared’ in some way, hermeneutically ready, to experience all those things in the way I did. But how? What exactly was it that I had brought there?” (p. 198).

Here, of course, my interpretive stance is no less speculative than the ones just considered; they simply square better with how we have come to think about the world. I am not trying to suggest that all of the different interpretive stances one might adopt are equally valid. Nor am I suggesting that the one I landed upon is arbitrary. On the contrary: everything I know about the world, at this particular point in time, at this particular place, points me in the very direction I am about to outline. And so I ask again: What exactly was it I had brought there?

First and perhaps foremost, I had brought a *world* to that trip to Berlin, a cultural horizon—or what Gadamer (1975) and others (e.g., Allan, 1993; Jauss, 1989; Shils, 1981) have referred to as *tradition*. This dimension of tradition—comprising those “implicit and unthematized . . . habits of the heart or mind or muscles” (p. 24) that ground our experience—was surely operative that day, conditioning what I saw and sensed.

There is no question but that I had also brought a good deal of concrete knowledge with me, “from books and movies and photographs and any number of other such things” (Freeman, 2002, p. 199), all of which seemed to have “activated the undercurrents,” as I had put it, of what I was witnessing. All of these had become part of my “memory,” and while second-hand, they surely served to condition some of what I saw and felt that day. “‘Memory,’ in this context,” thus emerges as “a curious amalgam of fact and fiction, experiences and texts, documentary footage, dramatizations, movies, plays, television shows, fantasies, and more” (p. 199).

Finally, for now, it seemed clear that my Jewish background needed to be figured into the equation too. As I indicated at the time, it was difficult to say exactly how. I don’t consider myself particularly religious. Nor did I know of family members who had suffered at the hands of the Nazis; all of my grandparents had landed in the United States before 1920, my parents were both born in the States, and, although we did have some relatives who perished at the hands of the Nazis (which I didn’t know at the time), I hadn’t come to Berlin with any discrete expectations of what I would find. Would I have responded the same way if I wasn’t Jewish? It’s impossible to say. But I would guess not. I therefore went on to question how this might have factored in. “Why, given my own ambivalences and uncertainties and hesitations” about my Jewish heritage, “had there been such a powerful encounter? Was it *despite* all these things? Was it *because* of them? Was it due to there being an admixture of identification and nonidentification, approach and avoidance, connection and dis-connection?” The answer: “I do not know” (p. 199), I said. What I did know was that there existed dimensions of my history and my identity about which I had been largely unaware.

This is where the idea of the “unconscious” enters the picture, which I use “not so much in the Freudian sense [of that which has been repressed, banished from consciousness] . . . but in a more explicitly *cultural* sense, having to do with those largely unrecognized and in turn uncognized aspects of our own histories that have been bequeathed to us by virtue of our status as historical beings of a specific sort” (2002, p. 201). More simply, what we are considering here are “those culturally rooted aspects of one’s history that have not yet become part of one’s story” (p. 193). As for the idea of the *narrative* unconscious, we encounter it “precisely during those moments when our own historical and cultural situatedness comes into view” (p. 201). My history comprises more than what I can consciously know and tell. Moreover, it is not to be restricted to that stretch of time between my birth and the moment of reflection. In a distinct sense, I am *born* into a history and carry it with me throughout the course of my life, often in ways unbeknownst to me. And when I come upon experiences that serve to jar this history loose and bring it into view, it can be quite startling and revelatory.

## Memory, Meaning, Mediation

Let me turn now to someone else’s story, someone for whom the narrative unconscious loomed particularly large. The person in question is Eva Hoffman, whose remarkable book *After Such Knowledge: Memory, History, and the Legacy of the Holocaust (2004)* does well to flesh out some of the ideas just introduced. As Hoffman notes early on in the book,

I had grown up with a consciousness of the Shoah from the beginning. My parents had emerged from its crucible shortly before my birth. They had survived, in what was then the Polish part of the Ukraine, with the help of Polish and Ukrainian neighbors; but their entire families perished. Those were the inescapable facts—the inescapable knowledge—I had come into. But the knowledge had not always been equally active, nor did I always want to make the inheritance defining.

Indeed, it was not until I started writing . . . that I began discerning, amidst other threads, the Holocaust strand of my history. I had carried this part of my psychic past within me all my life; but it was only now, as I began pondering it from a longer distance and through the clarifying process of writing, that what had been an inchoate, obscure knowledge appeared to me as a powerful theme and influence in my life. Until then, it had not occurred to me that I was in effect a receptacle of a historical legacy, or that its burden had a significance and weight that needed to be acknowledged. Now, personal memory appeared to me clearly linked to larger history, and the heavy dimensions of this inheritance started becoming fully apparent. (p. x)

It was made that much more apparent by the fact that most of those who had had first-hand experience of the Holocaust had died. So it was that Hoffman had

felt more and more palpably that the legacy of the Shoah was being passed on to us, its symbolic descendants and next of kin. We were the closest to its memories; we had touched upon its horror and its human scars. If I did not want to the ‘memory’ of the Holocaust to be flattened out through distance or ignorance, if I wanted to preserve some of the pulsing complexity I had felt in survivors’ own perceptions, then it was up to me. (pp. x-xi)

Hoffman thus “needed to reflect on [her] own and [her] peers’ link to that legacy, to excavate our generational story from under its weight and shadow—to retrieve it from that ‘secondariness’ which many of us have felt in relation to a formidable and forbidding past. In a sense,” she explains further, “I needed to address frontally what I had thought about obliquely: the profound effects of a traumatic history, . . . the kinds of knowledge which the Shoah has bequeathed to us, and the knowledge we might derive from it” (p. xi).

This meant exploring and interrogating the narrative unconscious, the “secondariness” at hand being explicitly linked to Hoffman’s membership in the “second generation.” On her account,

The second generation’s story is a strong case study in the deep and long-lasting impact of atrocity; and . . . children of survivors’ very personal transactions with the past are a strong clue to the problems we must grapple with if we would grasp the meaning and consequences of historical horror. In their mediated but immediate relation to the Holocaust, children of survivors have had to live out and struggle with some of the defining issues that follow from atrocity: the internal impact of gratuitous violence and the transmission of traumatic memories across generations; the emotional intricacies of dealing with victims of persecution and the moral quandaries implicit in dialogues with perpetrators; the difficulties of witnessing the pain of others and of thinking about tragic pasts; and the relationship of private memory to a broader understanding of history. (p. xii)

Needless to say, Hoffman’s story brings us a significant step closer to the Holocaust than my own. She wasn’t there either, so strictly speaking, “we who came after do not have memories of the Holocaust. Even from my most intimate proximity,” she continues, “I could not form ‘memories’ of the Shoah or take my parents’ memories as my own” (p. 6). At the same time, her awareness of their pain and suffering “created an unconscious, or preconscious, ethics, and . . . in this system, just as war was the ground of being, so pain was the ground of personhood” (p. 13). The result: she had “absorbed [her] parents’ unhappiness through channels that seemed nearly physical. The pain of their psyches,” she writes, “reverberated in my body almost as if they were mine” (p. 14). Hoffman also speaks of larger events, such as the Warsaw uprising, that reverberated in her as well. These “would become my meaningful history, the history it is urgent to know because it belongs to one’s life, because it shapes ancestral fate and one’s own sensibility” (p. 18). Such are “the paradoxes of indirect knowledge,” a knowledge that continues to “haunt” Hoffman and others who “came after”:

The formative events of the twentieth century have crucially informed our biographies and psyches, threatening sometimes to overshadow and overwhelm our lives. But we did not see them, suffer through them, experience their impact directly. Our relationship to them has been defined by our very “post-ness,” and by the powerful but mediated forms of knowledge that have followed from it. (p. 25)

Hoffman is not entirely comfortable framing things in the way she as. Indeed, she admits, “It did not occur to me to think of myself as a ‘child of Holocaust survivors’ for many of my adult years. Other threads of causality, influence, development seemed more important; or at least I gave them other names. . . . Identities are malleable and multidimensional,” Hoffman notes, “and I am reluctant to fix my own through reifying labels. And yet, we do not only define ourselves; we are also defined by our circumstances, culture, the perceptions of others and—perhaps most of all—the force of an internalized past” (p. 27).

The notion of “mediation” is central to Hoffman’s account: “those who have not lived through the Shoah received its knowledge, at this late date, through mediations—sometimes several layers of them. We view it no longer directly, but through memorials, artistic representations, literature, film” (p. 178). This is also true for those who *did* live through the Shoah; now that years have passed, they too view it—or at least some of it—through much the same mediations. This is in no way to diminish their experience or to render it problematically “impure.” The narrative unconscious is a region neither of purity nor impurity. Rather, it is a region of what I have referred to as “deep history” and, in turn, “deep identity” (see Freeman, 2010a, 2010b) and is thus an inevitable fusion of our own contemporaneous experience, which is itself mediated, and those more distant mediations that derive from without.

## The Burden of the Past

In order to round out the picture I have been painting in this essay, I want briefly to turn to one last case, one that deals not only with the ways in which one’s deep history is constitutive of one’s identity but the ways in which one may be implicated by a history far removed—in time if not in feeling—from one’s own life. I am referring here to the case of my colleague and friend Roger Frie, who several years ago shared with me his discovery that his beloved grandfather had in fact been a member of the Nazi party. As Frie asks in a recent (2014) article,

What does it mean to be caught in a web of history, to be part of a traumatic past over which we have no control? We are born into history and culture and it is through our family that we are connected to these larger dimensions of experience. Our families provide us with narratives, stories that enable us to make sense of what we see and that implicitly shape what we know and remember at any moment in time. These narratives are an integral part of who we are, yet are not consciously organized. They remain largely unconscious, a compass by which our lives are pre-reflectively organized. At some stage, or at some point in life, we may be able to reflect on certain aspects of the narratives we inherit, and begin to question them, thus revealing new ways of seeing the world around us. I say “may,” because a reflective, conscious understanding on our situation is never a given. . . . Initially at least, our place is made for us, unconsciously structured by the language we speak, the history we inherit, and the culture and traditions that constitute who we are. (p. 2)

Frie’s discovery was a painful one. “My grandfather was caring and kind, artistically gifted, and maintained a sense of humor in the face of the destruction and hardship wrought by war,” he writes. “Although I knew early on that my grandfather belonged to the side of the perpetrators, I was always relieved that his history, and by extension my own, was not one of perpetration” (p. 3). Upon making the discovery, through a photograph, that his beloved grandfather may have been closer to perpetrator status than had been assumed,

I found myself suddenly upended. My understanding of the narratives and memories by which I came to see the past had inalterably shifted. Knowledge of my grandfather’s identity as a Nazi meant that I was the inheritor of an indelibly tainted history, connected however directly or indirectly with the perpetration of unimaginably heinous crimes. The fact that my grandfather appeared to be a “minor Nazi” was hardly consoling. It was the support lent by the rank and file that enabled the perpetration of mass murder to unfold. No matter how much I might want to disown this history, it is a part of my past, a part of who I am. (p. 8)

Here, we begin to move more fully into the *moral* dimension of the narrative unconscious. Just as Hoffman had been something of a “victim” of the Holocaust, Frie, it would seem, was something of a perpetrator—not, of course, for the actual crimes that had been committed but for his own distanced stance in relation to them. As he puts the matter, “It seems to clear to me now that I had been dissociating historical facts as a way of maintaining intact memories of my grandfather and avoiding uncomfortable, even forbidden family discussions. I may also have been protecting my mother and her siblings by not speaking more directly to the possibility of my grandfather’s involvement” (p. 10). Judging by this statement, Frie had had some subliminal awareness of what had gone on. That, at least, is what he says now, looking backward. “When I initially reflected on the questions I posed,” Frie admits,

I found myself wavering about whether to fully engage the past. After all, my grandfather’s actions took place long ago, in an entirely differently time and place from my own. How was his political allegiance, his support of an immoral regime, possibly connected to me? In a purely chronological sense, I am one of those who “came after,” and as such bear no direct responsibility for what happened before me. But any attempt to erase, or relativize the meaning of history in this way, is surely motivated by a singular wish for an unburdened past, for memory of a grandfather that is free of conflict. (pp. 12-13)

And this is a wish, Frie recognizes, “that must ultimately fail” (p. 13).

When I initially heard Frie’s story, and saw the guilt and shame it had created in him, my initial impulse was to protect him, to do what I could to reassure him that he was a good man. As I put the matter back then (I was discussant for the first paper he gave following his discovery), the ultimate reference of the situation at hand was to the victims of the Holocaust themselves, to “the great mass of absent others whose call had gone unheeded. Roger seems to be hearing their call now,” I said, “sounding more loudly, perhaps, than ever before. The fact that he wasn’t there, the fact that he couldn’t be there, hurts. This bespeaks the depth of Roger’s own moral and ethical world. You should take some solace in this, Roger. The fact of your shame, and pain, is important” (Freeman, 2014a, p. 277).

I then went to address one last issue that seemed worth thinking about:

So much of what you’ve said in this excellent, provocative, courageous paper is about our belonging in and to history. As I know you know, I wouldn’t want to challenge any of it. But I would want to add something to it—something that can somehow accommodate your own shame and pain, your own sense that what went down, years ago, is a transcendent evil, one that is, at once, utterly and completely within history as well as beyond it. This, in turn, points in the direction of some sort of transcendent good—the very good that informs the judgments being made, however unconsciously.

Here, then, I shall just ask: What is the nature of this call you have heard? Where does it come from? And why does it speak with the urgency and intensity it does?

I ended my response (and the paper that was eventually published documenting it) right there.

## Thinking Otherwise About the Narrative Unconscious

Perhaps, as I bring this essay to a close, I can begin to answer these very difficult questions. This “call” is the call of the Other—in this case, other people, whose lives had been broken and destroyed by evil (see Freeman, 2012, 2014b). As for where it comes from, I can only conclude that it comes from somewhere either deep within us, perhaps some quasi-Jungian primordial history reverberating through the years, or, more likely, something outside of us, something Other, drawing us beyond ourselves, beyond our particular histories and cultures. As much as my own Jewish identity may have paved the way for that experience in Berlin, my own humanness may have been even more formative. Here, I am referring to my own confrontation with the sheer monumentality of suffering that was wrought during the Nazi era and the massive loss it left in its wake. As I had noted at the time, “it was as if there was a great hole in the middle of history. A part of us was missing from humanity. And a part of humanity was missing from us” (Freeman, 2002, p. 198). Those words still seem right to me, and they signal the existence of a dimension of human reality that is less easily contained by the idea of the narrative unconscious.

For Hoffman (2004) too, there was something excessive in her experience, something unable to be contained by any purely cultural-historical account. She recalls, for instance, her parents’ first communications, and how their “fragmentary phrases lodged themselves in my mind like shards, like the deadly needles I remember from certain fairy tales, which pricked your flesh and could never be extracted again” (p. 11). There were images too: “fields, trenches, pits of death, . . . barbed wire, skeletal figures, smoke, intimations of mass death.” According to Hoffman, “Every child has such images available right behind the eyelids.” And eventually, “through literature and film, through memoirs and oral testimony, these components of horror became part of a whole generation’s store of imagery and narration” (p. 12). This implies that there is a dimension of experience that *precedes* these various media and that allows them to take hold in the psyche. The result was “an unconscious, or preconscious, ethics,” grounded in pain. Some of this pain was surely her own. But its source was those *others*—her mother and father, most notably—who had felt this pain first-hand.

Roger Frie’s situation was, of course, very different, not least because he had been on the side of the perpetrators rather than the victims. As we observed, this yielded its own brand of pain. But it was a pain that issued from the depths of his conscience, his intense shame serving to signify, even if from a distance, his own felt implication in the horrific crimes of the past. “As much as I might want to step out of my historically defined position as a third-generation German,” he said at one point, “I am unable to” (p. 13).

Despite my inclination to console Frie on some level, essentially by reminding him that the very fact of his shame testified to his moral depth, I also had to say that, irrespective of what he might have suspected or even known about his grandfather’s potentially dastardly deeds, this “historically defined positon” was his to reconcile. In the end, it wasn’t clear to me how much it mattered what his grandfather had or hadn’t done, for the history at hand would be virtually the same in either case. Along the lines being drawn, it could be that Frie’s discovery simply served to concretize and thereby awaken heretofore unformulated thoughts and feelings that were part and parcel of his own cultural-historical experience as a German, living in the wake of catastrophe.

What I have come to see more clearly in my own continued reformulation of the idea of the narrative unconscious is the ethico-moral dimension. It’s not just that we belong to history and culture in ways unbeknownst to us. It’s that our way of belonging often seems to bear within it strong valuations regarding what is right and good—and wrong and evil. It is of course possible that these strong valuations are themselves products of history and culture, tied to the specific ways in which we are socialized and enculturated. This is surely part of it. But I have come to wonder whether these valuations also bespeak something beyond these processes, something that comes with the realities of being human and feeling the pull—and the priority—of the Other. So it is that I have come to speak not only of the narrative unconscious but the *ethical* unconscious. I don’t mean to suggest that this is some wholly different register of the unconscious. Indeed, it may be that the narrative unconscious, the ethical unconscious, the “classical” unconscious of psychoanalysis, and even that seemingly far-out notion of the collective unconscious are all of a piece, differentially distributed in accordance with our life circumstances and our varying degrees of awareness of what, in us, may be operative behind the scenes. As always, there is a much more to think about.
